# CD66b Overexpression and Loss of C5a Receptors as Surface Markers for *Staphylococcus aureus*-Induced Neutrophil Dysfunction

**DOI:** 10.1371/journal.pone.0132703

**Published:** 2015-07-15

**Authors:** Thomas Schmidt, Alva Brodesser, Norbert Schnitzler, Thomas Grüger, Kerstin Brandenburg, Jörg Zinserling, Josef Zündorf

**Affiliations:** 1 Department of Dermatology, Philipps-Universität of Marburg, Marburg, Germany; 2 Federal Institute for Drugs and Medical Devices, L2 Biosafety Laboratory, Bonn, Germany; 3 Gesundheitsamt des Kreises Düren, Düren, Germany; 4 Federal Institute for Drugs and Medical Devices, Biostatistics Unit, Bonn, Germany; University of Tokyo, JAPAN

## Abstract

Neutrophil granulocytes constitute the main component of innate immunity in the clearance of bacterial infections. However, during systemic inflammation, immunoparalysis may occur resulting in neutrophil dysfunction. This study presents a new *in vitro* model for analyzing the dysfunction of human peripheral blood neutrophils resulting from the interaction with *Staphylococcus aureus* components in whole blood. After induction of a massive complement activation by *S*. *aureus* supernatant, the neutrophils exhibit a reduced phagocytic capacity resulting in a dramatic reduction of the antibacterial activity similar to that of neutrophils isolated from septic patients. The number of phagocytozing neutrophils is drastically reduced as well as the phagocytic capacity designated by a significantly lower number of ingested microbes. This dysfunction correlates with the loss of complement component 5a receptor 1 from the neutrophil cell surface and can be further characterized by a C5a-induced CD66b overexpression. The presented *in vitro* model offers a new platform for preclinical testing of immunosuppressive drugs and delivers new information for the understanding of neutrophil dysfunctions under the conditions described.

## Introduction

Granulocytes belong to the first line defense of the innate immune system when pathogenic microorganisms like *S*. *aureus* have invaded the host. Colonization is a known risk factor for infections which range from local skin infections over deep tissue infections to sepsis [1]. A systemic spread of the pathogen leads to overshooting immune reactions accompanied by immune dysfunction. Despite the availability of antimicrobial drugs, *S*. *aureus* is one of the most frequent and lethal causes of bloodstream infection. This emphasizes the need for improving our knowledge about its mode of action on the immune system [2]. Neutrophils are the most prominent granulocytes that are recruited to sites of infection. Many cell types and humoral systems including coagulation components contribute to the manifestation of systemic inflammation [3]. Aside from a profound immune activation e.g. induced by the massive release of proinflammatory cytokines [3–5], the effects of severe immune suppression (especially the immune paralysis of peripheral blood neutrophils) play a major role in defective immune regulation [6]. Neutrophil dysfunction is characterized by a strong decrease in phagocytic activity [7, 8], oxidative burst [7], a delayed apoptosis [9] and a transmigration deficiency [10–12]. Due to the impaired antimicrobial activity, the patients have an increased risk of acquiring further infections.

Some reports have described a change in the expression of the activation markers CD66b [10, 13] and CD11b [10, 13, 14] during sepsis, but a correlation between neutrophil dysfunction and CD66b:CD11b expression has not been observed. CD66b is a GPI-anchored glycoprotein of the carcinoembryonic antigen (CEA) family and is located in the specific granules [15]. It is only known to be expressed on human granulocytes and on the granulocytes of two primate species [16] and is associated with the aggregate formation of human neutrophils [17]. CD11b is a β_2_ integrin which is located mainly in the secretory vesicles and is a central component in the transmigration process [18]. After activation, the neutrophils exhibit elevated levels of both molecules on the cell surface and in this context both molecules are well-known markers for cell activation [18, 19].

Animal models have already delivered important aspects towards understanding the development of sepsis but in some cases these data cannot be transferred to humans. For example, in an endotoxin-based rat model of sepsis the increase of the C5a receptor (C5aR) 1 is in opposition to a significant loss of this molecule in human sepsis patients [20, 21] [22]. For studies addressing the involvement of molecules like CD66b which are completely absent in experimental animals like mice or rats, data from the human organism are essential. Due to the insufficient datasets describing the defective innate immune response in humans, a detailed analysis of the human conditions including the kinetics of the events has not been possible so far [23]. Thus, the development of new models focusing on immune suppression under septic conditions is required in addition to clinical data. Here, we are presenting an *in vitro* model for analyzing the immunological disorder of human peripheral blood neutrophils induced by *S*. *aureus*. In this study, *Staphylococcus aureus* supernatant (SaS) serves as a tool to initialize the special cell activation that leads to pathophysiological effects similar to the effects described in sepsis. The generated neutrophil phenotype is characterized by a loss of phagocytic activity and a strong decrease of the phagocytic capacity similar to cells isolated from septic humans [5]. This effect is accompanied by an increase of aggregate formation and a dramatic reduction in the antimicrobial activity of whole blood. Furthermore, the affected cells exhibit a special activation phenotype which can be characterized by a strong decrease in C5aR 1 and a C5a-induced special CD66b:CD11b molecule ratio on the cell surface which might serve as a new marker for intravascular neutrophil dysfunction. This model may also deliver new aspects for understanding the immunoparalysis of neutrophils which can be observed in sepsis.

## Material and Methods

### Bacteria, bacterial culture and collection of bacterial supernatant

Procedures were performed as described previously [17, 24]. For production of *Staphylococcus aureus* supernatant (SaS), three different *Staphylococcus aureus* strains from the same sequence type 9 but from various spa types were used (for further information see reference [15]). These different SaS are referred as SaS A, SaS B and SaS C in the following text. After a 18 h culture in IMDM (Invitrogen, Carlsbad, CA, USA; Lot: 561098) bacteria were pelleted by way of centrifugation, and the cell-free supernatant was passed through a 0.2μm-pore filter (Whatman, Springfield Mill, UK; Lot: 84833) to remove bacterial residues. In order to avoid strong dilution effects in the cell samples, the fluid volume of each overnight SaS was reduced by using a 9-kDa protein concentrator (Pierce, Waltham, MA, USA; Lot: 137628B) following the manufacturer´s protocol. For functional assays we used *Staphylcoccus aureus* ATCC52359.

### Blood donors

Blood samples were collected from healthy donors between 21 and 59 years of age. Prior to each blood donation, volunteers were excluded in the case of infections, immune disorders, severe diseases or intake of medication. In addition, each blood sample was analyzed by ACT8 (Beckmann Coulter, Brea, CA, USA) for the parameters WBC, RBC, Hct and Plt. Blood samples not within the normal range of any parameter were not used in the experiments. The coagulation cascade was inhibited by adding heparin (10 IU/ml; ratiopharm, Ulm, Germany). The Ethics Committee of the Ärztekammer (medical association) Nordrhein accepted the study plan and the information leaflets for the participants. Written informed consent was obtained from each study participant.

### Neutrophil activation and C5a receptors

Neutrophils were treated prior to the functional studies as described previously [17]. For the activation of neutrophils in whole blood the stimuli fMLF (2 x 10^−8^ M; Sigma-Aldrich, St. Louis, MO, USA) and SaS (1:100) were used. SaS was collected as described above. After a 25-fold concentration step, the dilution factor 1:100 leads to a final concentration of bacterial substances which corresponds to 25% of the overnight culture supernatant. After blood collection either fMLF, SaS or IMDM were added, the blood was mixed briefly and incubated at 37°C. After 15 minutes (timepoint A 0 min) a fixed volume of suspension was taken and stored immediately on ice. Then the bacteria were added to the rest of the sample and the functional assays were continued (see below).

In experiments including the C5a receptor antagonist W-54011 (2 μM; Calbiochem, Nottingham, UK) the respective substance was preincubated in blood for 5 min (37°C) prior to stimulation.

### Detection of human anaphylatoxin C3a, C4a and C5a in blood plasma

Plasma was obtained from blood samples which were incubated for 15 minutes with IMDM, SaSA and fMLF as described above. The BD Cytometric Bead Array (CBA) Human Anaphylatoxin Kit was used to quantitatively measure anaphylatoxin C3a/ C3a desArg, C4a/ C4a desArg and C5a/ C5a desArg protein levels in the plasma samples at timepoint A0. All steps were performed according to the instructions of the kit and as described previously [25]. In short, the capture beads were incubated with defined standard concentrations of each anaphylatoxin and with the experimental samples. After washing, the incubation with anti-C3a, -C4a and -C5a PE conjugated antibodies allows the formation of sandwich complexes (bead—analyte—fluorescent antibody). The resulting complexes were measured and identified by fluorescence characteristics of both beads and detector reagent. Cytometric analysis was performed by FACSCalibur and data were analyzed by way of the FCAP array software (BD Biosciences, New Jersey, USA).

### Phagocytosis and oxidative burst

In order to assess phagocytosis, *Staphylococcus aureus* ATCC52359 was cultured overnight (18 h / 37°C) in IMDM. One ml of this overnight culture was forwarded to 19 ml of fresh medium and incubated under the same conditions unless an optical density of 0.2 to 0.6 (590 nm) had been reached. Bacterial cells were harvested by centrifugation, washed with NaCl (0.9%) and incubated with bis-carboxyethyl-carboxyfluorescein-pentaacetoxy-methylester (BCECF/AM, molecular probes; final concentration 1 μmol / liter) for 30 min at 37°C in 1 ml NaCl (0.9%) as described previously [26]. Non-fluorescent BCECF/AM diffuses into the bacteria and is cleaved by cytoplasmic esterases to yield the fluorescent membrane-impermeable BCECF that remains trapped in vital cells. In order to achieve a bacteria:granulocyte ratio of 10:,1 the number of bacteria was determined after staining using a Multisizer 3 (Beckmann Coulter, Brea, CA, USA) and the amount of white blood cells in whole blood was calculated by ACT8 (Beckmann Coulter, Brea, CA, USA / number granulocytes = factor 0.7 x number of white blood cells). The labeled bacteria and blood samples (with and without the respective pretreatment) were mixed gently and incubated at 37°C for different timepoints. The analysis of phagocytosis by flow cytometry includes the parallel determination of CD66b and CD11b receptor expression and covers measurements at 3 min, 6 min, 10 min, 15 min, 30 min, 60 min, 120 min and 180 min.

In order to determine the production of reactive oxygen intermediates (ROI) such as O_2_ˉ, OHˉ or H_2_O_2_ during the oxidative burst, unlabeled bacteria were incubated in heparinized blood under identical conditions as described for the phagocytosis assay but in the presence of dihydrorhodamine (DHR; Molecular Probes, Eugene, OR, USA). DHR was added to a final concentration of 10 mg/l. DHR is freely permeable, localizes in the mitochondria of PMN and emits a bright green fluorescent signal upon excitation by blue light (488 nm) after oxidation by H_2_O_2_ and O_2_ˉ to rhodamine 123 during the respiratory burst. The analysis of the oxidative burst by flow cytometry includes the parallel determination of CD66b and CD11b receptor expression and covers measurements at 3 min, 6 min, 10 min, 15 min, 30 min, 60 min, 120 min and 180 min.

### Bacterial killing

The killing assay was performed as described previously [27]. After harvesting and washing, the bacteria were diluted in NaCl (0.9%). Following pretreatment of blood samples (see above) the cells were incubated with the bacteria in a ratio of 10:1 (bacteria / granulocytes) as in the phagocytosis and oxidative burst assay. Initially and after 2 hours and 3 hours of rotation at 37°C, the suspension was plated in serial dilutions (10^−2^ to 10^−4^) onto HHD agar plates. After 18 hours (37°C), the numbers of bacterial colonies were calculated and expressed as the difference between log_10_ colony-forming units per milliliter indicating the amount of killed bacteria.

### Staining protocol, flow cytometry analysis and gating parameters

Staining procedure and preparation of samples were performed as described previously [17]. The stained and fixed cells were analyzed by the flow cytometers FACSCalibur (Becton Dickinson, Heidelberg, Germany) and Image Stream^X^ (Amnis, Seattle, WA, USA). The threshold for positive staining was set according to isotype controls. Neutrophils were gated as CD66^+^CD11b^+^ population or as CD11b^+^ in combination with their forward and side scatter characteristics. Gating and determination of mean fluorescence was done using FlowJo software (FlowJo, Ashland, OR, USA) for the FACSCalibur analysis and by IDEAS software (Amnis, Seattle, WA, USA) for the ImageStream^x^ data. For consideration of the non-specific activation of neutrophils caused by the *in vitro* conditions, some of the flow cytometry data are analyzed and presented as the relative increase with respect to the negative control (fixed as value 1). For receptor staining, the following fluorochrome-labeled antibodies and isotype controls were used: Mouse anti-human CD66b FITC, BD Pharmingen (Lot: 62302); Mouse anti-human CD66b PE, BioLegend (Lot: B140697); Mouse anti-human CD11b APC, eBioscience (Lot: E11648-1634); Mouse anti-human CD88 PE, Biolegend (Lot: B136933); Mouse anti-human C5L2 PE, Biolegend (Lot: B152518); IgG2a PE, Biolegend (Lot: B166017); IgM FITC, BD Pharmingen (Lot: 46139); IgG1 APC, eBioscience (Lot: E10294-1633).

### Neutrophil imaging and aggregation

The combination of ImageStream^x^ and IDEAS software allows determination of the mean fluorescence of cell populations with simultaneous photographing of each cell in the bright field and in all relevant fluorescence channels. In the present study, all pictures were taken using a 40 x objective. Neutrophil gating and the separation of single cells and larger aggregates from cell doublets was performed as described previously [17]. For determination of the amount of bacterial events co-located with neutrophils after phagocytosis, single cells were separated from doublets by way of several gating steps as described above. With the spot-count function (number of events located in one image or cell), the number of BCECF-positive events per cell or events per doublet was calculated.

### Statistics

Experiments were conducted several (mostly 6–10) times, each with a different blood donor. The flow cytometric analysis included evaluation of a minimum of 8,000 PMN and results were pooled for statistics. Data are presented as mean values and standard deviations for descriptive statistics if not specified otherwise (SPSS 17.0, IBM SPSS Statistics, Armonk, NY, USA). Normal distribution was checked visually from distributions and by way of the Shapiro-Wilk W test. One-way and two-way repeated measures ANOVA were used and pairwise comparisons were performed with an LSD test. As comparisons are considered exploratory, no adjustment for multiplicity was performed. P values < 0.05 are considered significant.

## Results

### SaS-induced phagocytic dysfunction

**Fig 1:**
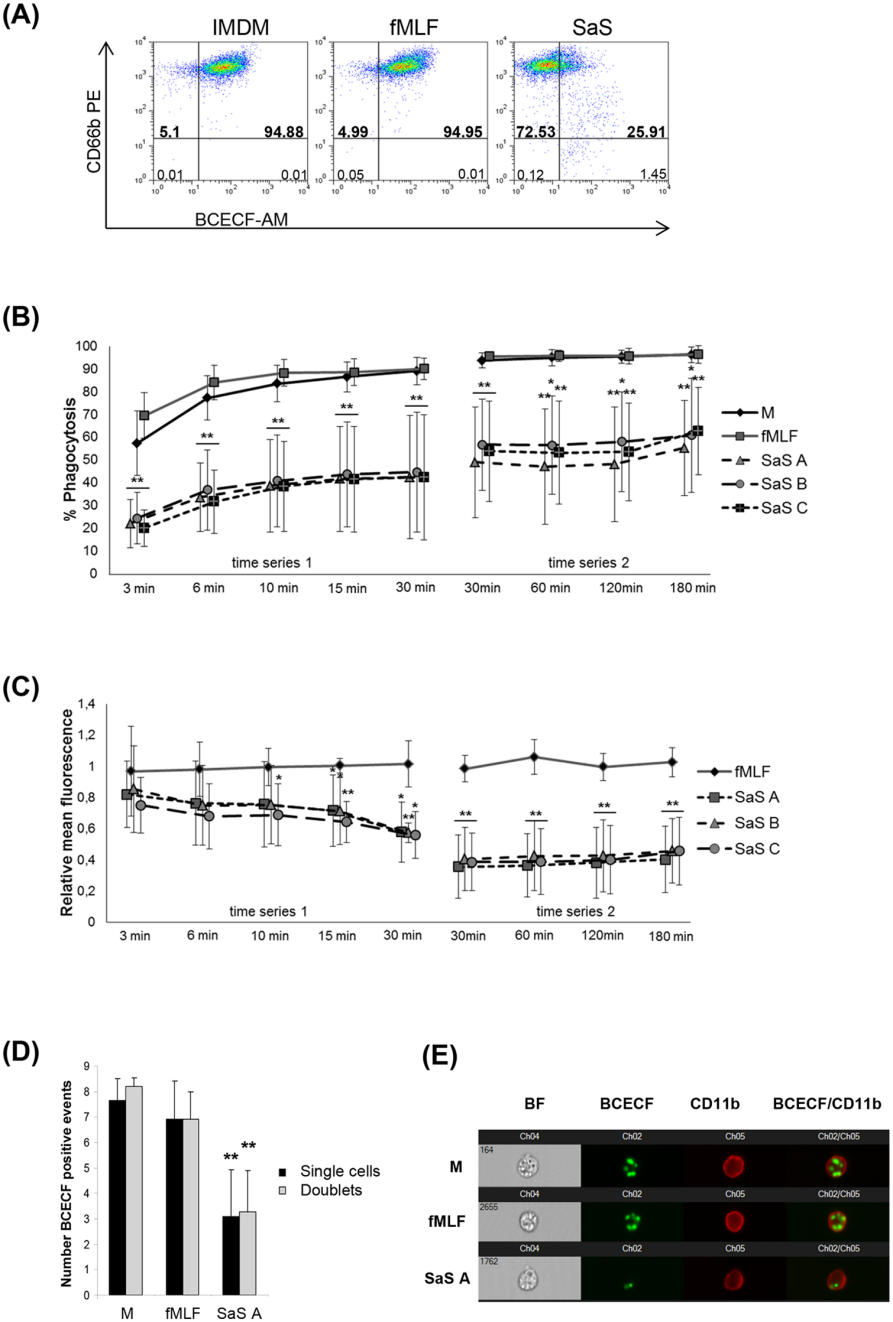
Phagocytosis of peripheral blood neutrophils after stimulation with SaS. A 15 min preincubation with M (media control), fMLF (2 x 10^-8^M) or SaS A-C (1%) was performed prior the functional studies. (A) Phagocytosis of BCECF-labelled ATCC52359. Analyzed timepoint: 30 min. X-axis is presenting the BCECF intensity (absolute values), y-axis is presenting the CD66b expression (absolute values). (B) Number of phagocytozing cells (% of whole cell population). Analyzed timepoints: 3, 6, 10, 15, 30 min (experimental series 1; n = 6) and 60, 120, 180 min (experimental series 2; n = 7). (C) BCECF-fluorescence intensity of the BCECF-positive cell populations. Analyzed timepoints: 3, 6, 10, 15, 30 min (experimental series A; n = 6) and 60, 120, 180 min (experimental series B; n = 7). Values are presented as the relative mean of fluorescence in comparison with the negative control. (D) Number of phagocytozed bacteria of single cells and cell doublets. Analyzed timepoint: 30 min. The number of ingested bacteria was calculated per cell which means that values obtained for doublets were divided by 2. Analysis was done by using Image Stream^x^ (Amnis, Seattle, WA, USA) in combination with the Spot-Count-function of the IDEAS-software (for more detail information see Material and Methods). n = 6. (E) Image selection of neutrophil single cells after phagocytosis (30 min) and preincubation with M, fMLF or SaS. All photos were taken with a 40 x objective (Image Stream^x^, Amnis, Seattle, WA, USA), analyzed by IDEAS software (Amnis, Seattle, WA, USA) and illustrated in a channel series, including the BF (bright field) mode; the fluorescence channels imaging the staining for ATCC52359 (BCECF / Channel 2), CD11b (PE / channel 3) and an overlay of channel 2 and channel 3. Error bars indicate ± SD *P < 0.05 **P < 0.01 compared to fMLF.

The impact of *S*. *aureus* exoproteins on neutrophil function was analyzed by way of the phagocytosis assay. In contrast to the negative control (media control, M) and the stimulation with fMLF, preincubation with SaS resulted in a strongly reduced number and percentage of phagocytozing neutrophils (Fig 1A). Thirty minutes after addition of the bacteria there were only 26% phagocytozing cells in comparison to 96% in the control groups (M, fMLF). This effect was consistent for the three SaS types used (SaS A, B, C). It is noteworthy that the extent of phagocytozing cells after SaS treatment varied highly among the individual experiments. These experiments included phagocytic rates between 10% and 80%. The amount of BCECF-positive cells was analyzed over a long range of time (3, 6, 10, 15, 30, 60, 120, 180 min). At the beginning of the kinetics (3 min—10 min) we observed identical reduction of phagocytozing cells irrespective of their significantly reduced amount after SaS treatment (Fig 1B). Already 3 min after addition of the bacteria, a 50% reduction of phagocytozing cells in the SaS-treated samples compared to the control groups could be observed. Despite the difference in the number of phagocytozing cells this effect indicates similar kinetics of bacteria ingestion. The first peak in the number of phagocytozing cells was reached after 10 min of incubation. Here, the analysis showed a phagocytic rate of approx. 85%- 95% for M and fMLF and 20%– 40% for the three SaS. This amount was consistent for M and fMLF until 180 min, whereas we observed a slight but not statistically significant increase of phagocytozing cells between 30 min and 60 min and further between 120 min and 180 min for the three SaS.

In addition to the number of cells that ingested BCECF-labelled bacteria, we also determined the mean fluorescence for the BCECF-positive cell population to measure the phagocytic capacity (relative number of ingested bacteria). The results demonstrate a negative impact of SaS on the relative amount of ingested bacteria (Fig 1C). Thirty min after addition of the bacteria, the BCECF-positive neutrophils of the SaS groups showed a 50% less intense fluorescence compared to the control populations. In relation to the negative control we observed a constant decline in mean fluorescence from 3 min until 30 min. In comparison to the media control the fMLF-stimulated cells were not affected in their ingestion capacity over time.

In order to further evaluate whether the lower intensity of BCECF fluorescence is based on a decreased fluorescence intensity of the dye, the number of BCECF-positive events per cell was calculated by way of the spot-count function of the IDEAS software. The results confirmed the fluorescence data (see above). In comparison to the control groups which were able to ingest 8 (M) or 7 (fMLF) bacteria per cell for the SaS-treated fraction, the results show a bacterial ingestion rate of 3 bacteria per cell which means that the phagocytic capacity was reduced by more than 50% (Fig 1D and 1E). Additionally, we were not able to detect any differences in the phagocytic capacity between single cells and cell doublets.

In conclusion, the results confirmed an SaS-induced phagocytic dysfunction of neutrophil granulocytes which was characterized by a dramatic decrease in the number of phagocytozing cells in combination with a markedly reduced phagocytic capacity of each single cell.

### Impairment of oxidative burst and bacterial killing

**Fig 2:**
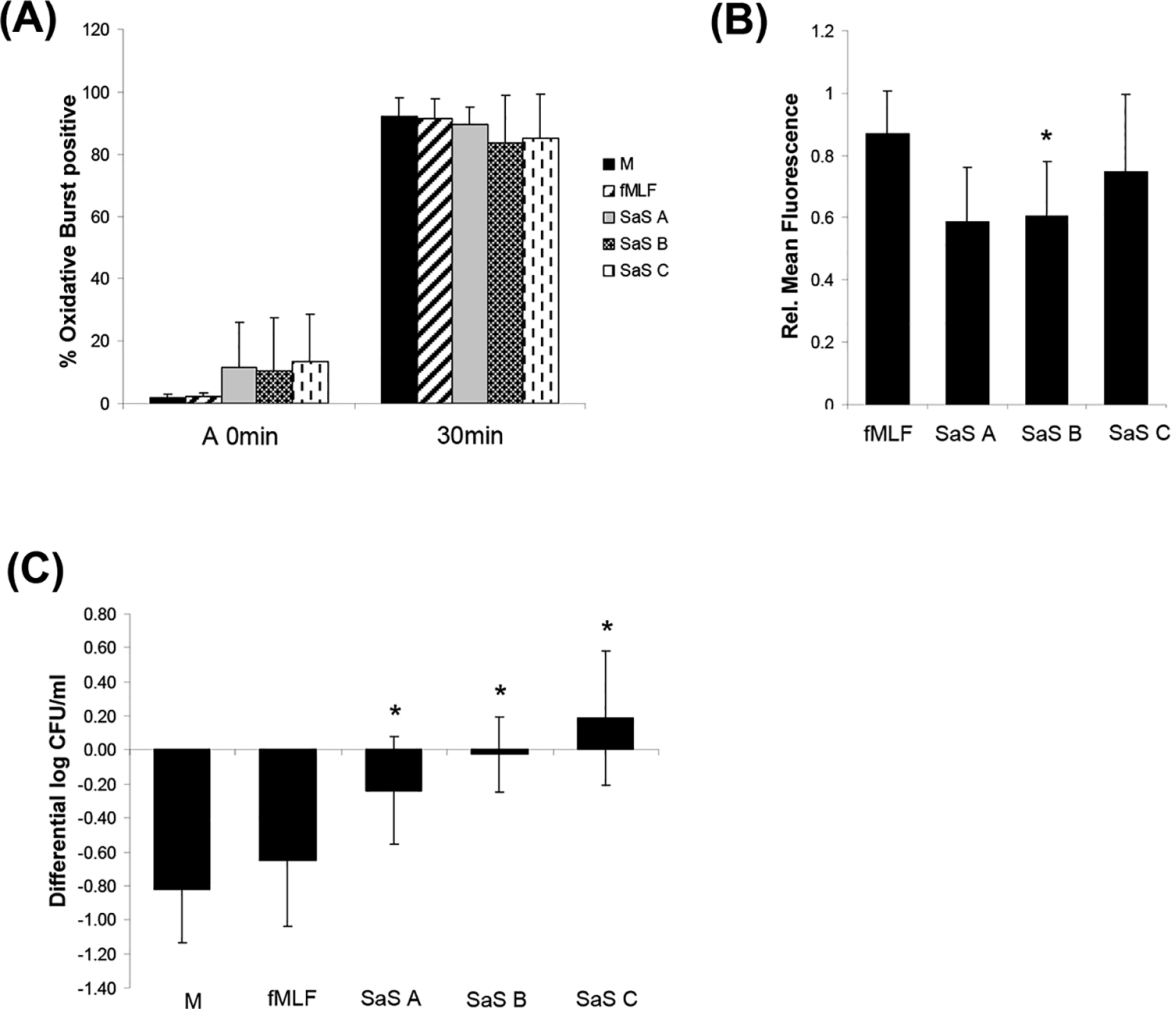
Oxidative burst in human neutrophils and whole blood bactericidal assay after stimulation. A 15 min preincubation with M (media control), fMLF (2 x 10^-8^M) or SaS A-C (1%) was performed prior the functional studies. (A) The oxidative burst was determined at timepoint A 0 min and after 30 min. (B) Values are presented as the relative mean of fluorescence in comparison with the negative control after 30 min. Error bars indicate ± SD *P < 0.05 compared to fMLF. (C) Whole blood bactericidal assay after stimulation. 15 min preincubation with M (media control), fMLF (2 x 10^-8^M) or SaS A-C (1%) was performed prior the functional studies. ATCC52953 was incubated with whole blood and the decline of vital bacteria was determined at a defined timepoint. Analyzed timepoint: 180 min. The decline of vital bacterial cells was illustrated on the y-axis as the change in log CFU / ml of the respective inoculum (for more detail information see Material and Methods). n = 6. Error bars indicate ± SD *P < 0.05 **P < 0.01 compared to fMLF.

In order to test whether the impairment of phagocytosis is compensated e.g. by an elevated release of anti-microbial substances, the generation of reactive oxygen species was determined. The oxidative burst was measured in the identical experimental setting as described above including the time points A 0 min and 30 min. It was shown that the oxidative burst was not affected as strongly as the phagocytosis. In contrast to the media control and the fMLF sample in some experiments the cells already showed a slightly increased fluorescence level after preincubation with SaS (A 0 min) indicating a starting oxidative burst. It was not possible to find any correlation between these phenomena and other effects e.g. a pronounced phagocytosis inhibition. 30 min after addition of the bacteria for all groups we observed a rate of “burst”-positive cells of almost 100% (Fig 2A). The analysis of these cell populations revealed that the SaS-treated cells show a 40% lower fluorescence intensity compared to the media control (Fig 2B).

Due to the fact that we were not able to distinguish between “phagocytozing” and “not-phagocytozing” cells we do not know whether the discrepancy between the fluorescence intensity was caused by the partial inhibition of phagocytosis because the oxidative burst is induced particularly after ingestion of pathogens.

In order to further test for an increased activity of other immune cells (like monocytes), the anti-microbial capacity of whole blood was determined by a killing assay. After 180 min of incubation in the media control and the fMLF sample, the amount of live bacteria was markedly reduced whereas in the SaS group we only observed stagnancy (SaS A, SaS B) or even an increase of in the number of viable bacteria (SaS C). The results demonstrate that the SaS-induced neutrophil dysfunction led to a strong reduction of the anti-microbial activity of whole blood (Fig 2C).

### Development of a special CD66b:CD11b ratio

**Fig 3:**
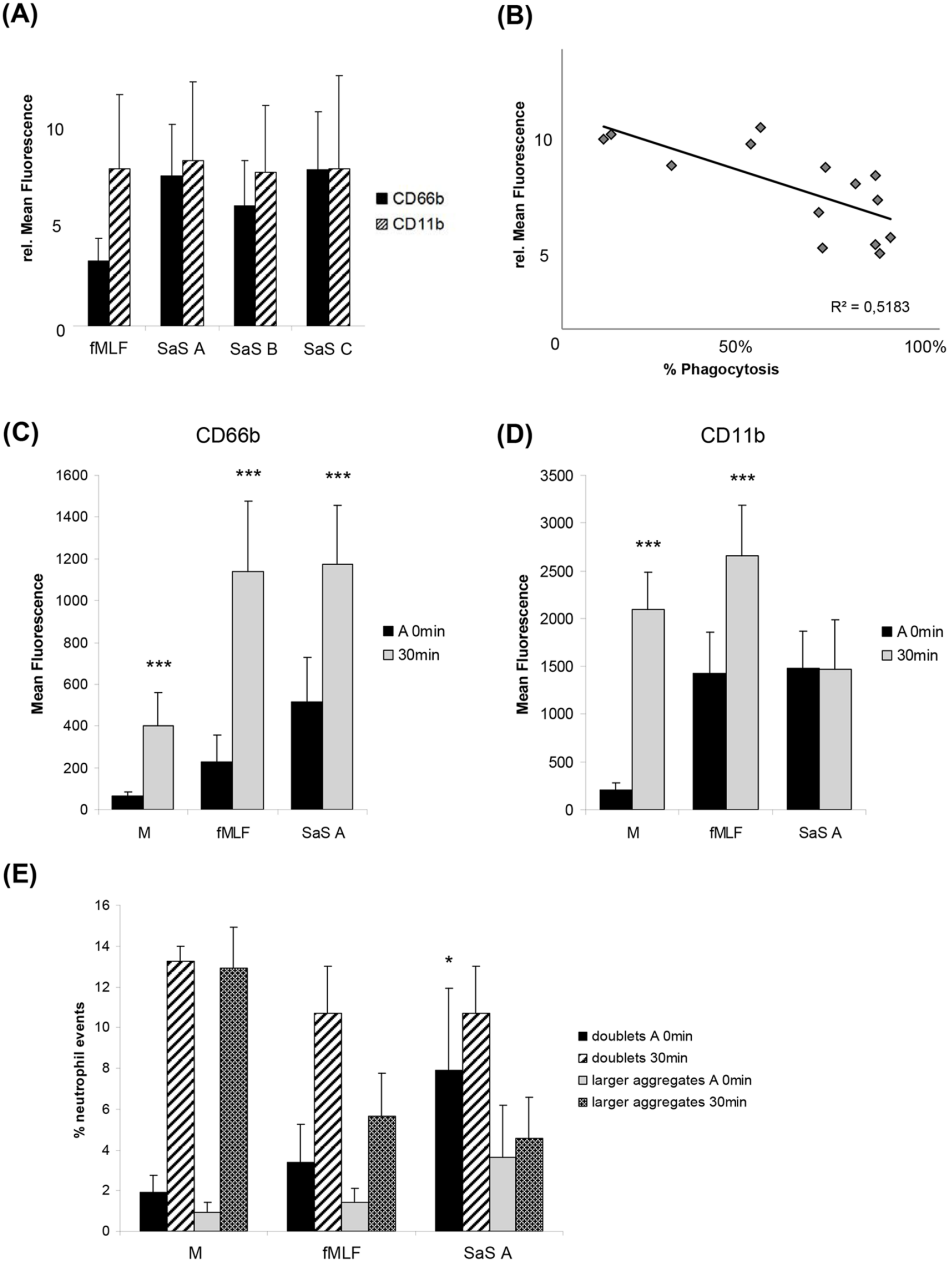
CD66b and CD11b expression, and aggregate formation. A 15 min preincubation with M (media control), fMLF (2 x 10^-8^M) or SaS A-C (1%) was performed prior the functional studies. The timepoint after 15 min of incubation is defined as A 0 min. (A) CD66b and CD11b expression of human neutrophils after incubation with fMLF and SaS A-C. Analyzed timepoint: A 0min. Values are presented as the relative mean of fluorescence in comparison with the negative control (n = 20). (B) Correlation of SaS A stimulation-induced CD66b expression at timepoint A 0 min and resulting %phagocytosis after 30 min incubation with bacteria. (C) CD66b expression on human neutrophils. Analyzed cell population / timepoint: whole cell population / A 0 min (M, fMLF, SaS A); phagocytosis assay—cells with ingested bacteria / 30 min (M, fMLF, SaS A). Values are presented as the relative mean of fluorescence in comparison with the negative control. n = 13. (D) CD11b expression on human neutrophils. Analyzed cell population / timepoint: whole cell population / A 0 min (M, fMLF, SaS A); phagocytosis assay—cells with ingested bacteria / 30 min (M, fMLF, SaS A). Values are presented as the relative mean of fluorescence in comparison with the negative control. n = 13. Error bars indicate ± SD ***P < 0.001 compared to A 0 min. (E) Aggregate formation of human neutrophils. Analyzed timepoints: A 0 min, 30 min / after addition of bacteria. Values are presented as the percentage of whole neutrophil population. n = 6. Error bars indicate ± SD *P < 0.05 compared to fMLF.

For further characterisation of the phenotype of the neutrophil granulocytes after SaS treatment (A 0 min) we examined the surface expression of CD66b and CD11b after staining with molecule-specific monoclonal antibodies and subsequent flow cytometry analysis. Fifteen minutes after addition of SaS A, B and C the relative amount of CD66b and CD11b on the cell surface was strongly increased (Fig 3A). In comparison to fMLF, the cells exhibited a similar amount of CD11b but a highly elevated CD66b expression. Thus, we observed an fMLF-induced molecule ratio of approximately 1:2 whereas for the SaS samples the overexpression of CD66b lead to a special CD66b:CD11b molecule ratio of almost 1:1 (always referring to media control). For correlation of the CD66b molecule expression with the phagocytosis data we examined the relative upregulation of CD66b and the corresponding phagocytosis rate after preincubation with SaS A. The results demonstrated that the molecule can be directly correlated to phagocytic activity (Fig 3B). In the experiments with the most pronounced inhibition of phagocytosis, CD66b was more densely expressed as compared to the experiments where the phagocytic activity was hardly affected.

### CD66b and CD11b expression and aggregate formation during phagocytosis

The development of the CD66b:CD11b expression pattern was also examined after the addition of bacteria. The analysis focused on differences between the cell population without bacteria (BCECF-negative) and cells that had already finished phagocytosis / ingested bacteria (Fig 3C and 3D). After 30 min of incubation for the BCECF-positive fraction the results revealed a similar expression of CD66b after stimulation with fMLF and SaS (absolute fluorescence intensity). Despite the different amount of CD66b on the cell surface at the starting point (A 0 min), the cells in all samples were capable of inducing a strong increase in molecule expression that led to a similar amount of surface molecules 30 min after addition of bacteria (Fig 3C).

However, with regard to the CD11b expression the results revealed a strong discrepancy between the SaS-induced and the media/fMLF-induced molecule patterns. Thirty minutes after addition of bacteria for the media control/fMLF samples we observed a strong increase of CD11b on the cell surface (Fig 3D). In contrast, the preincubation with SaS lead to the perpetuation of a constant CD11b level identical to the molecule density at the starting point (A 0 min). Neither the pronounced changes in the environmental conditions caused by addition of the bacteria nor a phagocytic activity showed any effect on the level of CD11b expression. The extent of aggregate formation (doublets and larger aggregates) under the varying conditions was calculated by the IDEAS software as described above. In the setting without bacteria, the results showed a significantly greater number of cell doublets and larger aggregates after SaS stimulation compared to media control and fMLF. Particularly in the samples stimulated with media control and fMLF, the addition of bacteria led to a steep increase of both aggregate types indicating a direct connection between phagocytosis and aggregate formation (Fig 3E). In contrast to this, the number of SaS-induced aggregates was not influenced by the changing environmental conditions. After 30 min we observed a similar amount of cell doublets in all samples but a significantly elevated level of large aggregates in the media control.

### Complement activation and loss of C5a receptors

**Fig 4:**
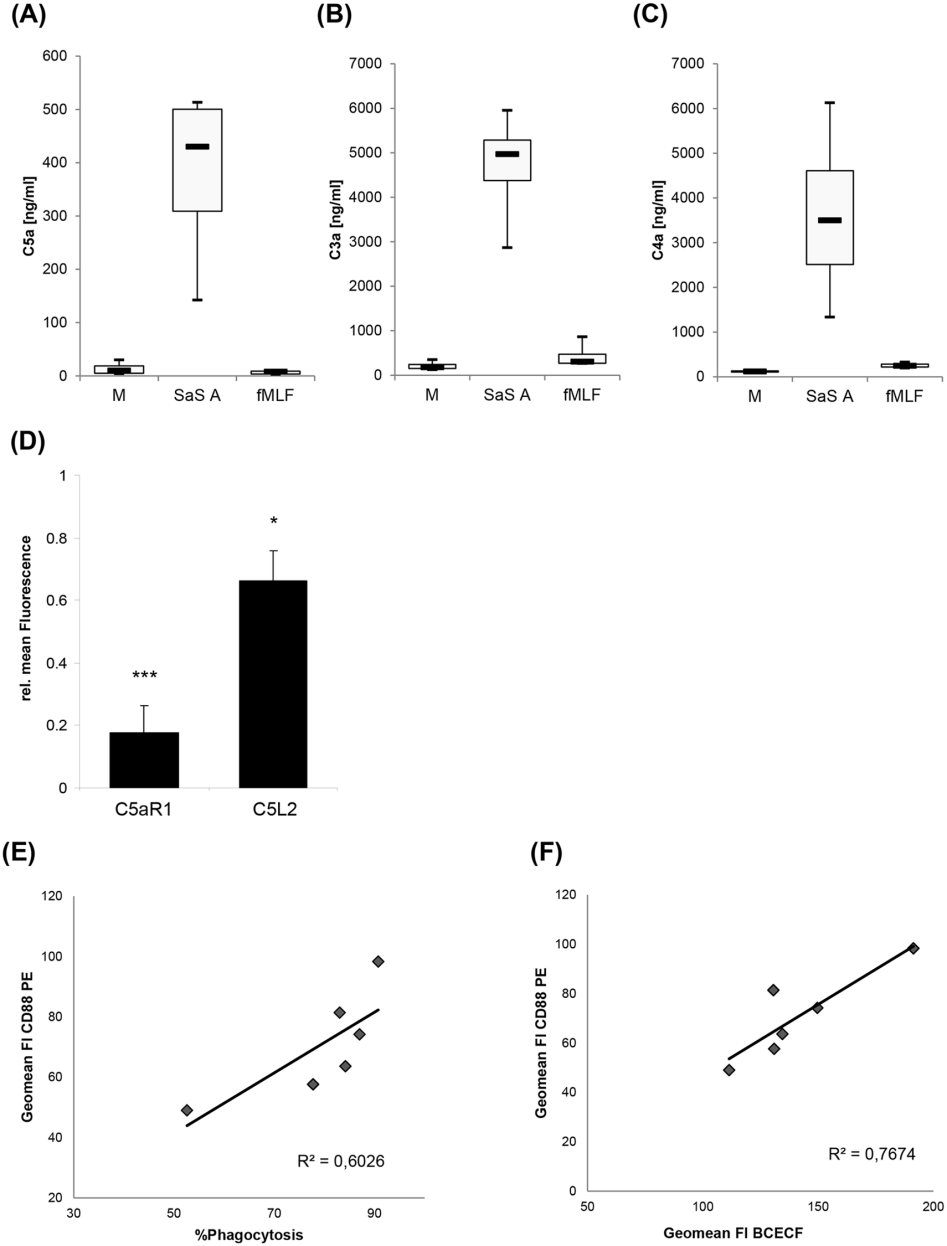
Complement activation and C5a receptor expression. Concentration of complement components (A) C5a / C5a desArg, (B) C3a / C3a desArg and (C) C4a / C4a desArg after 15 min stimulation with M, SaS A and fMLF in whole blood. Data were analyzed with a non-parametric method using Friedman rank ANOVA for dependent samples due to the low number of cases and are presented as median with upper and lower quartile; error bars indicate minimal and maximal value, respectively. After stimulation, one median out of three for each complement component is considered significantly different. n = 4. (D) Expression of C5aR1 and C5L2 after stimulation with SaS A at timepoint A 0 min. n = 6. Error bars indicate ± SD *P < 0.05 ***P < 0.001 compared to media control. (E) Correlation of C5aR1 expression with % of phagocytozing PMN after stimulation with SaS A and (F) correlation of C5aR1 with BCECF fluorescence intensity after stimulation with SaS A, indicating the amount of internalised bacteria.

In order to analyze complement activation after the different stimuli, the concentration of anaphylatoxins after stimulation with control medium, SaS A and fMLF in whole blood was determined after 15 min of incubation. In contrast to stimulation with SaS A, stimulation with M and fMLF resulted in relatively low ranges/levels of C5a, C4a and C3a. The concentration of all three complement factors after incubation with the bacterial supernatant was increased up to 20fold (Fig 4A, 4B and 4C). Two receptors on neutrophils are known to bind to C5a. In order to examine the role of the C5a receptors C5a R1 and C5L2 in the development of the special CD66b:CD11b molecule ratio and the phagocytic dysfunction, the surface expression of C5aR1 and C5L2 was analyzed. The expression of both C5a receptors was strongly reduced (Fig 4D). Fifteen minutes after addition of SaS the C5L2 was reduced by30% and C5aR1 was reduced by more than 80% as compared to media control. After SaS stimulation, the fluorescence intensity of the anti-C5aR1 antibody correlated positively with the amount of phagocytozing PMN (Fig 4E) and with the phagocytic capacity of neutrophils, depicted as the fluorescence intensity of BCECF (Fig 4F).

**Fig 5:**
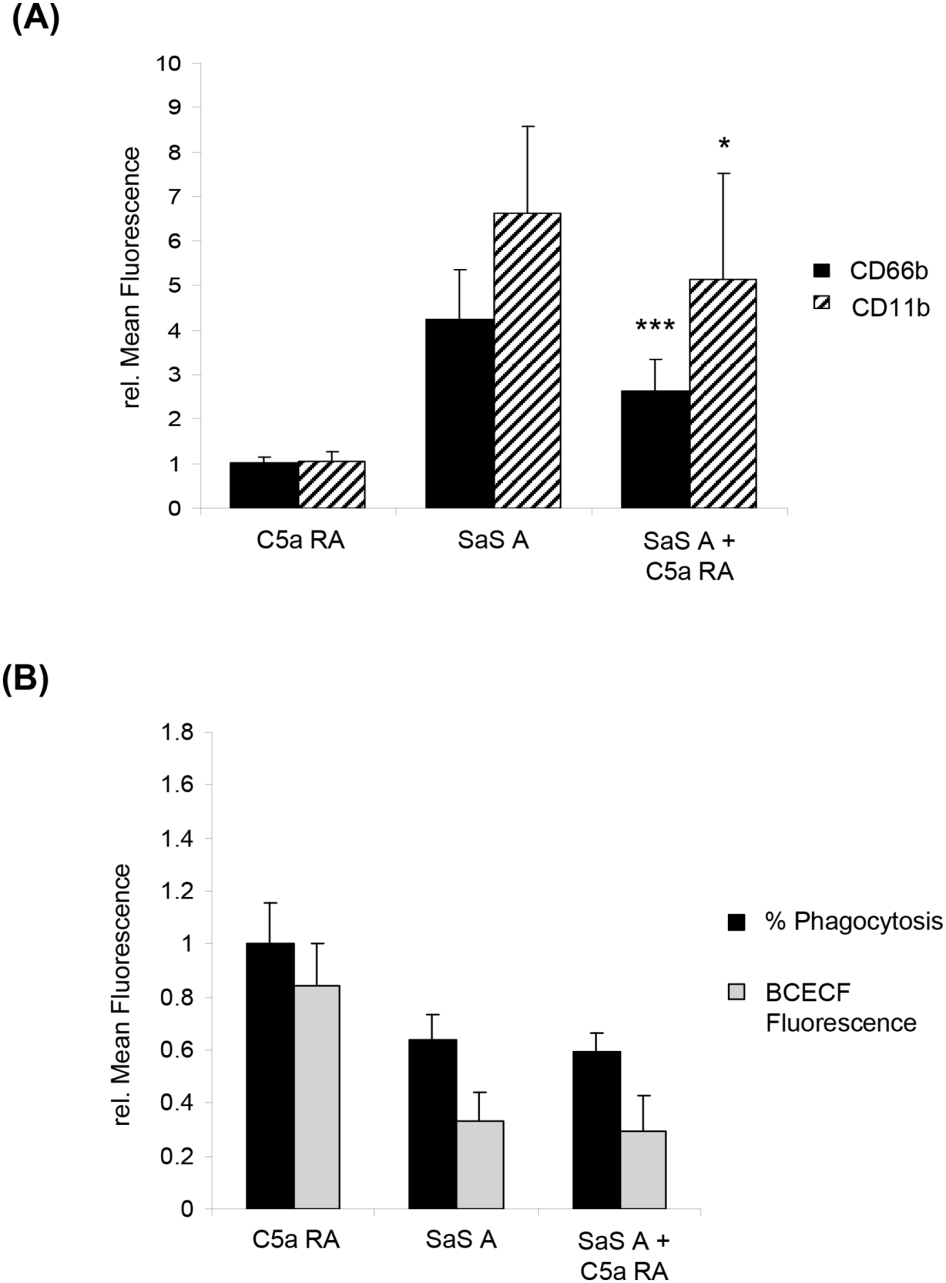
Impact of C5aR1 antagonist W-54011 on CD66b:CD11b expression and phagocytosis. A 15 min preincubation with M (media control) or SaS A (1%) was performed prior the functional studies. The timepoint after 15 min of incubation is defined as A 0 min. (A) Impact of W-54011 (= C5a RA) on the CD66b:CD11b expression. Analyzed timepoint: A 0 min. Values are presented as the relative mean of fluorescence in comparison with the negative control. C5a RA n = 5; SaS A, SaS A + C5a RA n = 7. Error bars indicate ± SD *P < 0.05 ***P < <0.001 compared so SaS A. (B) Impact of W-54011 (C5a RA) on phagocytosis. “% Phagocytosis”—number of phagocytozing cells compared to whole population, “mean fluorescence BCECF”–mean fluorescence intensity of BCECF-positive cell population (phagocytic capacity). Values are presented as the relative mean of fluorescence in comparison with the negative control. %Phagocytosis: n = 8; Mean Fluorescence BCECF: n = 11. Abbr: C5a RA = C5aR1 antagonist W-54011.

Additionally, we performed assays with and without the specific C5aR1 antagonist W-54011. CD66b and CD11b expression increased after stimulation by SaS A (Fig 5A). Under media control conditions no impact of the antagonist on the CD66b:CD11b expression, phagocytic capacity and number of phagocytozing cells could be observed (Fig 5A and 5B). However, we observed a strong influence of the SaS-induced phenotype compared to the control without the antagonist after preincubation with the C5aR1 antagonist. This change could be characterized by a significant decrease of CD66b and CD11b expression after A 0 min (Fig 5A). Although samples including the C5aR1 antagonist showed a reduced receptor expression at A 0, representing the timepoint when bacteria were added, the antagonist did not have an impact on the SaS-induced phagocytic dysfunction: the phagocytic capacity and the number of phagocytozing cells and fluorescence of internalized bacteria remained constant (Fig 5B).

## Discussion

Systemic inflammation and sepsis are complex pathophysiological disorders and many components are involved in the generation of a septic milieu [3]. However, as already mentioned in the introduction, there is still a need for *in vitro* models which simulate distinct features of these syndromes, including neutrophil paralysis. In our experimental model, we were able to generate a milieu to elucidate the dysfunction of peripheral blood neutrophils under such conditions. In order to simulate these physiological conditions whole blood samples were preincubated with SaS and the bacteria were added subsequently. The incubation with SaS induced a massive activation of complement and lead to a neutrophil dysfunction similar to that observed in septic patients [5]. This malfunction is mainly based on a strong decrease of phagocytic activity and capacity. Phagocytosis of pathogens represents the first step in the central anti-microbial activities phagocytosis/oxidative burst/bacterial killing of neutrophils. The ingestion of bacteria is directly linked to the initiation of oxidative burst and the production of other anti-microbial substances which are responsible for a successful bacterial killing. Thus, it is likely that the decrease of oxidative burst and bacterial killing is a consequence of the defective phagocytosis. In septic patients, this immune disorder is described with a poor outcome of disease [8, 28]. In the model, the dysfunction seems to be a rather stable phenomenon because there is no improvement of phagocytosis until 120 min after the first bacterial contact.

Inhibition of phagocytosis by SaS might be induced by SaS-specific virulence factors. In the last study, we provided an overview of virulence genes in the supplement which shows that strain A has CHIPS and SCIN expressing genes, whereas this is not the case for strain B and C [17]. Nevertheless, all three SaS inhibit phagocytosis of bacteria in the same manner. Ko et al. describe a specific staphylococcal protein that interferes directly with phagocytosis by binding opsonizing C3b with its n-terminus and fibrinogen with its c-terminus, thus preventing bacteria from iC3b-mediated ingestion by neutrophils. This protein which is called extracellular fibrinogen-binding protein (Efb) is found in the genetic profile of all strains used for the SaS. The direct correlation of complement activation (C5a) and neutrophil dysfunction could also be explained by Efb because the action of this molecule is highly dependent on complement activation. If Efb is the central factor protecting bacteria by way of fibrinogen-binding, the whole neutrophil population should be influenced by this phenomenon and a homogeneous inhibition of phagocytosis would be expected. This might indicate a bacterially independent process as recently described by van den Berg et al. [29]. They postulate that a C5a-induced release of serine proteases which cleave C5a receptors on the cell surface is responsible for a complement-induced neutrophil dysfunction. Our data do not support van den Bergs theorem, as the inhibition of C5a receptors did not improve the phagocytic dysfunction in our system; however, inhibition of C5a R1 was able to abrogate the CD66b overexpression. Another cause for the inhibition of phagocytosis might be based on the change of the CD11b surface receptor expression. After incubation with SaS, we observe a direct relation between a suppressed CD11b boost and reduced phagocytosis. In view of the fact that CD11b as a complement receptor (iC3b) represents a central phagocytosis-supporting molecule [30], this effect is supposed to contribute to the decreased phagocytic function. It is noteworthy that the bacterially induced boost of CD66b expression is not affected by SaS. This indicates two autonomous signal transduction pathways for CD11b and CD66b expression. In neutrophil dysfunction there are indications of the involvement of the molecule PI3K [5, 8]. As PI3K represents a central signaling pathway component in the CD11b expression [31, 32], this might be one reason for the blockade of the CD11b boost. This means that the release of the specific granules which contain the CD66b molecule would be based on a PI3K-independent pathway.

The “dysfunction phenotype” after preincubation with SaS is characterized by a loss of C5a receptor 1 (C5aR1) and partly 2 (C5L2), an increased CD66b:CD11b ratio and a strong increase of neutrophil doublets and larger aggregates. The elevated aggregate formation as a reason for the strongly reduced phagocytosis rate can be excluded by calculation of the ingested bacteria per cell. Here, we observed an almost identical number of bacteria in single cells and cells in a doublet formation. This reveals that despite aggregate formation, the neutrophils seem to exhibit a consistent phagocytic activity. Furthermore, it is possible that the measured events are newly formed aggregates of single cells after ingestion of bacteria because the neutrophil aggregate formation in whole blood after SaS treatment is a dynamic process [17].

The loss of C5aR1 and C5L2 represents a marker for complement activation [33, 34]. After SaS treatment the activation of the complement cascade was confirmed by the increase of C3a, C4a and C5a. Gram-positive substances like peptidoglycan and lipoteichoic acids, especially when combined, are already identified potent inducers of the pathophysiological conditions described [35, 36]. This is the main reason for the decision to apply SaS as a “gram-positive mix”, instead of using single bacterial substances. Furthermore, a replacement of plasma by cell culture media totally abrogates the activating effects of SaS in whole blood [17] and the usage of the C5aR1 antagonist W-54011 strongly affects the expression of the activation markers CD66b and CD11b (see below). Additionally, the almost identical behavior of the cells after treatment with supernatant of three different *S*. *aureus* strains indicates an indirect and relative non-specific activation mechanism like stimulation by pro-inflammatory complement components. This shows that the described phenotype is probably caused by the massive complement activation. Complement molecules, especially C5a, are well-known inducers of neutrophil dysfunction during sepsis [37]. The C5a level in blood correlates with the severity of sepsis [38] and the blockade of this molecule in a rat model of cecal ligation puncture (CLP)-induced sepsis leads to a restoration of cell functions like chemotaxis, H_2_O_2_ production and phagocytosis [39]. In our model, the loss of C5aR1 directly correlates to phagocytic dysfunction and loss of phagocytic capacity. The complement-induced immune paralysis represents a serious problem during manifestation of sepsis because it is often associated with a worst outcome [8, 28]. Therefore, an effective monitoring of the immune functions is essential. As mentioned before, the loss of C5aR1 on the cell surface represents a biomarker for a high complement exposure [33] and therefore the reduced presence of C5aR1 on the neutrophil surface is also a sign of complement-induced neutrophil dysfunction in septic patients [8]. In a rat model of a CLP-induced sepsis, the loss of C5aR1 is associated with a poor prognosis [40].

After binding of C5a at C5aR1, the receptor is removed from the cell surface by intracellular translocation [33]. Unfortunately, down-regulation of C5aR1 can also occur after exposure to other inflammatory mediators like IL-8 and TNF [41]. Thus, additional markers for the identification of a complement-induced dysfunction are required. In our model, besides the reduction of both C5a receptors C5aR1 and C5L2, the neutrophils exhibit a changed CD66b:CD11b ratio. In contrast to fMLF (CD66b:CD11b-ratio approx. 1:2) the cells show a CD66b:CD11b ratio of nearly 1:1 (relative to media control) at 15 min after addition of SaS. This difference is caused by an overexpression of CD66b. Interestingly, a correlation between CD66b overexpression and phagocytic malfunction revealed a direct connection of the two phenomena. In experiments with a minor dysfunction, the cells show a lower receptor expression in contrast to experiments where the phagocytic capacity of neutrophils was strongly affected. This indicates a direct connection between the CD66b receptor and the severity of neutrophil paralysis and might deliver a new marker for a complement-induced dysfunction and even a marker to determine the intensity of inhibition of neutrophil function. For proof of this thesis, more experiments and adequate datasets of septic patients would be required.

As mentioned before, the special molecule ratio is caused by an overexpression of CD66b. CD66b is located in the specific granules and therefore it represents a surface marker for the exocytosis of these cell organelles. The release of the granules is initiated by activating soluble mediators like cytokines or complement factors like C5a [42]. C5a represents the complement molecule with the highest pro-inflammatory potential [43]. Most of the functional effects of C5a are due to C5aR1, especially the pro-inflammatory responses including the degranulation processes [22]. To further investigate the role of C5aR1 and to find a potential connection between phagocytic dysfunction and the parallel release of the specific granules, the C5aR1 antagonist W-54011 was used. In contrast to our previous findings [17] due to a modified protocol used in this study (double amount of C5aR1 antagonist combined with a shorter incubation period) we observe a strong influence on the molecule expression when the C5aR1 antagonist is applied. The inhibition of C5aR1 leads to a significant reduction of CD66b and CD11b expression indicating a lower cell activation status and a successful block of the granule exocytosis. These results are in agreement with our previously published data where a substitution of blood plasma by cell culture media prevents an SaS-induced CD66b overexpression [17]. Interestingly, the C5aR1 antagonist failed to reveal any effects on the phagocytic dysfunction. It seems that there is a phenomenological but no functional relationship between the special molecule ratio and the defective phagocytosis. This might be an indication that the repressed expression of the phagocytosis-supporting receptor CD11b rather than the CD66b overexpression is responsible for the lack of phagocytic activity. In another *in vitro* model based on C5a-stimulated isolated human neutrophils, a C5aR1-blockade prevents an impairment of phagocytosis [5]. This reflects again that there is a strong divergence between the “behavior”of isolated neutrophils and neutrophils still interacting with components of whole blood, as in our set-up. Even the use of a different C5a antibody may alter the results, whereas we used a specific C5aR antagonist which prevented the C5aR from binding to its natural ligand.

Granulocyte-macrophage colony-stimulating factor (GM-CSF) might be another therapeutic agent in the treatment of impaired phagocytosis. In several studies, including critically ill patients, restoration and preservation of neutrophil functions was observed after a GM-CSF treatment [5, 44].

In summary, using our set-up we could reconstruct several features which are also observed in neutrophil granulocytes from septic patients. Despite the limitations of *in vitro* settings dealing with this topic our study presents a new powerful tool for analyzing the “behavior” of blood leukocytes under the described conditions and delivers new aspects of the neutrophil immune paralysis.
